# Organic Anion Transporting Polypeptide 3A1 (OATP3A1)-Gated Bio-Orthogonal Labeling of Intracellular Proteins

**DOI:** 10.3390/molecules28062521

**Published:** 2023-03-09

**Authors:** Krisztina Németh, Zsófia László, Adrienn Biró, Ágnes Szatmári, Gergely B. Cserép, György Várady, Éva Bakos, Csilla Özvegy-Laczka, Péter Kele

**Affiliations:** 1 Chemical Biology Research Group, Institute of Organic Chemistry, RCNS, Magyar Tudósok Krt. 2., H-1117 Budapest, Hungary; 2 Molecular Cell Biology Research Group, Institute of Enzymology, RCNS, Magyar Tudósok Krt. 2., H-1117 Budapest, Hungary; 3 Membrane Protein Research Group, Institute of Enzymology, RCNS, Magyar Tudósok Krt. 2., H-1117 Budapest, Hungary

**Keywords:** organic anion transporter polypeptide 3A1, large Stokes shift fluorescent probes, live cell bio-orthogonal modification, intracellular labeling, cell permeability, self-labeling enzyme tags, confocal microscopy, super-resolution microscopy, flow cytometry

## Abstract

Organic anion transporting polypeptides (OATPs) were found to readily deliver membrane impermeable, tetrazine bearing fluorescent probes into cells. This feature was explored in OATP3A1 conditioned bio-orthogonal labeling schemes of various intracellular proteins in live cells. Confocal microscopy and super-resolution microscopy (STED) studies have shown that highly specific and efficient staining of the selected intracellular proteins can be achieved with the otherwise non-permeable probes when OATP3A1 is present in the cell membrane of cells. Such a transport protein linked bio-orthogonal labeling scheme is believed to be useful in OATP3A1 activity-controlled protein expression studies in the future.

## 1. Introduction

Fluorescence microscopy techniques have emerged as an inevitable tool to image biomolecules or to study biomolecular processes in live cells [1,2,3]. Highly specific labeling techniques that combine synthetic biology with bio-orthogonal chemistry allow the visualization of a wide range of intracellular structures enabling the imaging of biological processes at high spatial and temporal resolution with minimal perturbation of the host organism. These methods rely on the prior specific bio-orthogonalization of the biomolecule of interest. For example, modification of proteins with fusion tags (e.g., SNAP, CLIP or HALO) or by genetic code expansion represent excellent means for the highly specific incorporation of bio-orthogonal functions (e.g., *trans*-cyclooctenes, or cyclooctynes) that are selectively reacted with fluorescent or fluorogenic markers carrying a complementary bio-orthogonal function (e.g., tetrazine or azide) [4,5,6,7,8,9,10]. In the context of live cell labeling schemes, membrane permeability of the probes is a requisite. Several strategies exist to render impermeable fluorescent cores with excellent photophysical features permeable [11,12,13]. Sometimes, however, a disadvantage such as impermeability can turn into an advantage, as we have recently shown in connection with a member of the organic anion transporting polypeptide (OATP) family. In our seminal paper, we reported our findings on membrane impermeable, zwitterionic fluorescent probes carrying a sulfonate function that were found to be potent substrates of human OATP3A1 [14,15,16]. Indeed, the probes were readily transported into HEK-293-OATP3A1 cells allowing functional investigation and the screen of drug interactions of the OATP3A1 transporter. Not only did these impermeable fluorophores turn out to be the first sensitive fluorescent probes for testing OATP3A1 substrate/inhibitor interactions; these results also highlighted the undiscovered potential of transport protein-conditioned delivery of probes into live cells. OATPs encoded by SLCO genes play a central role in the transport and distribution of xenobiotics and a wide variety of endogenous substrates [17]. OATPs are recognized as major determinants of the absorption, distribution, excretion and toxicity (ADME-Tox) properties of clinically important drugs [18]. Such a critical role of OATPs in drug disposition processes, therefore, has placed these membrane proteins in the focus of research both in academia and in the pharmaceutical industry. The better understanding of OATP-controlled processes enables us to answer clinical questions raised by the pharmaceutical industry in the context of pharmacotherapy. Of the 11 OATPs, some are thoroughly investigated, while the role of others remains less understood. For instance, the various functions of liver-specific OATP1B1 and OATP1B3 have been intensively investigated and our knowledge of their participation in the hepatic clearance of endobiotics (e.g., bile acids, bilirubin or steroid hormones) or drugs is well understood [19,20,21,22]. At the same time, we have very limited knowledge of other OATP family members. OATP3A1, responsible for the transport of estrone-3-sulfate (E1S), prostaglandins E1 and E2, thyroxine and vasopressin, is one of the least studied OATPs, even though it is widely expressed in the human body [23]. Its presence in several important barrier tissues such as the choroid plexus, the blood–ocular barrier, the lactiferous ducts of the breast and the Sertoli and germline cells of the testes is confirmed both by mRNA analysis and protein expression studies [24]. Furthermore, increased expression of OATP3A1 can be linked to certain cancers such as tumors of the liver and breast [23,25,26]. While many members of the OATP family are renowned drug transporters, drug interactions of OATP3A1 have yet to be thoroughly investigated. Not long ago, the OATP3A1 encoding gene (SLCO3A1) was identified as a novel Crohn’s disease-associated gene [27]. Functional studies suggest that expression of OATP3A1 promotes the activation of the NF-κB transcription factor mediating inflammatory processes, which consequently induces increased activation of ERK and JNK phosphorylation, resulting in more intense and protracted NF-κB activation in intestinal epithelial cells. These suggest that a more thorough understanding of OATP3A1 is highly desirable. However, the number of tools enabling functional studies of OATP3A1 is limited, mainly due to the lack of appropriate assays or methods that allow specific recognition of OATP-controlled intracellular processes.

## 2. Results and Discussion

**Figure 1:**
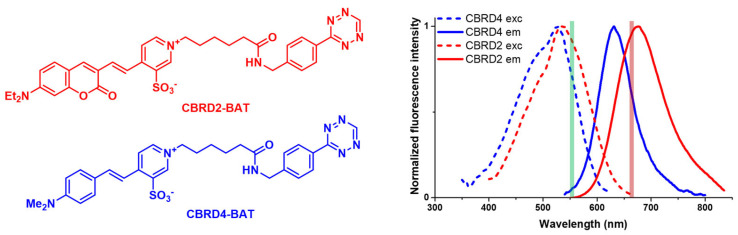
Structures, excitation and fluorescence spectra of dyes **CBRD2-BAT** and **CBRD4-BAT** and wavelength of the excitation and depletion lasers (green and red lines, respectively).

In a preceding study, we have demonstrated that these aforementioned membrane-impermeable large Stokes shift fluorescent cores readily transported by OATP3A1 possess retained spectral properties when modified with a tetrazine motif (benzylaminotetrazine, BAT) as bio-orthogonal handle [28] and allow low concentration and fast labeling reactions with extracellular POIs of live cells tagged with a cyclooctyne (bicyclo[6.1.0]non-4-yne, BCN) as the bio-orthogonal reaction partner. The probes were suitable to image-labeled structures with confocal and super-resolution (STED) microscopy. Therefore, we intended to use these BAT-modified probes (**CBRD2-BAT** and **CBRD4-BAT**) in the present study (Figure 1).

**Figure 2:**
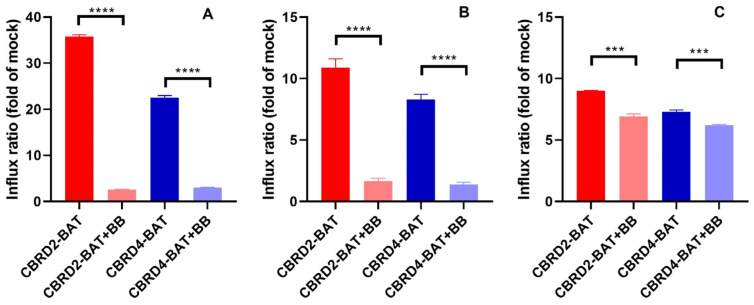
Influx ratio of dyes **CBRD2-BAT** (10 µM) and **CBRD4-BAT** (10 µM) in Uptake pH 5.5 (**A**) Uptake pH 7.4 (**B**) and in complete DMEM (**C**) into HEK-293-OATP3A1 compared to mock cells. Influx was determined as (mean RFU–relative fluorescence unit) with flow cytometry and inhibited with benzbromarone (BB) at 20 µM and 80 µM concentrations in Uptake buffer and DMEM medium, respectively. Significance of differences was estimated by t-test, **** <0.001; *** 0.004.

To this end, we first needed to confirm that these BAT-modified probes are similarly recognized by OATP3A1. Since transport activity of several OATPs, including OATP3A1, is activated by acidic extracellular pH, we tested the uptake of these probes by OATP3A1 overexpressing HEK-293 cells (HEK-293-OATP3A1) (Figure S1) in Uptake buffer pH 5.5 [14,15,16]. In addition, to better mimic cell culture conditions, uptake was also studied in FBS-complemented (complete) DMEM medium (pH 7.4). To our delight, modification of the probes with the tetrazine motif did not have any detrimental effects on the transport process of the zwitterionic probes when the Uptake buffer pH 5.5 was used. Consequently, flow cytometry analysis indicated a 36- and 22-fold increase of fluorescent intensity for **CBRD2-BAT** and **CBRD4-BAT**, respectively, compared to mock cells (Figure 2A). In complete DMEM medium, which is more suitable for cells, especially for longer time periods, a moderate 9.0- and 7.3-fold increase in fluorescence intensity in OATP3A1-overexpressing cells was observed (Figure 2C). We also assessed the selectivity of OATP3A1 in the transport of the **CBRD-BAT** probes. Thus, a specific OATP-inhibitor, benzbromarone (BB), was applied in 20 µM concentration, leading to a ca. 90% decrease of transport in experiments in Uptake buffer pH 5.5 (Figure 2A). When complete DMEM and 80 µM BB was used, only a 15–23% decrease in dye uptake could be observed, which can be attributed to the albumin binding of BB and the consequently lower free concentration of the inhibitor [29]. When albumin-free Uptake buffer was used at pH 7.4, the influx efficiency was similar to that measured in DMEM but, on the contrary, effective inhibition (83–85% with 20 µM BB) of probe uptake was observed (Figure 2B). These results prove that mainly the pH, and not the composition of the applied media, affects transport activity and, on the other hand, gives indication that FBS was responsible for the decreased inhibitory effect of BB.

We have also assessed the influx kinetics of the probes in Uptake buffer pH 5.5 and in complete DMEM (ESI Figure S2). The K_M_ values obtained in Uptake buffer were 7.2 µM and 45 µM for **CBRD2-BAT** and **CBRD4-BAT**, respectively, whereas K_M_ values in DMEM were 2.9 µM and 50.2 µM for **CBRD2-BAT** and **CBRD4-BAT**, respectively. The K_M_ values of the probes followed a similar tendency to the probes in our preceding study (29 and 192 µM [14]); however, the affinity of OATP3A1 for present tetrazine modified probes was observed to be higher.

We evaluated the effects of the buffers and the probes on cellular viability. Incubation of HEK-293-OATP3A1 and mock cells in Uptake buffer pH 5.5 for 15 min did not have any reportable effects, while co-administration of Uptake buffer and **CBRD** probes indicated a slight but not negligible decrease of viability (ESI Figure S3). The toxic effects were more pronounced in the case of **CBRD2-BAT**. On the other hand, the changes in viabilities remained within error in DMEM at 3 µM **CBRD2-BAT** or 10 µM **CBRD4-BAT** even with prolonged incubation times (i.e., 30 min), which underlines the importance of the buffer used. Although transport kinetics do not differ substantially between the two buffers (Uptake and DMEM), for viability considerations, DMEM medium was selected for live cell labeling studies.

Following these preliminary studies, we were eager to see whether HEK-293-OATP3A1 cells expressing bio-orthogonalized intracellular proteins can be labeled with these non-permeable tetrazinylated probes (**CBRD2-BAT** and **CBRD4-BAT**). To this end, HEK-293-OATP3A1 and HEK-293 mock cells were transfected with a selection of plasmids coding for various intracellular proteins (nuclear envelope protein, LaminA; histone protein, H2B; lysosomal protein, Lamp1; structural protein, Vimentin; and mitochondrial protein, TOMM20) fused at C-termini with self-labeling HaloTag. Bio-orthogonalization of the proteins was effected by treatment of the transfected cells with a cyclooctynylated HaloTag substrate, HaloBCN (3 µM, 60 min) (ESI Scheme S1).

**Figure 3:**
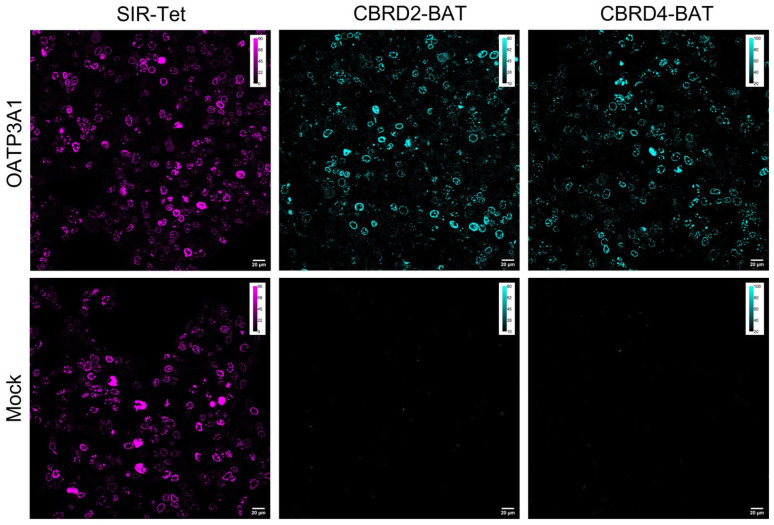
Confocal microscopy images of LaminA-HaloTag expressing OATP3A1 positive and negative (mock) cells pretreated with HaloBCN (3 µM, 60 min) and treated with non-permeable fluorescent dyes **CBRD2-BAT** (3 µM, 30 min) and **CBRD4-BAT** (10 µM, 30 min) in complete DMEM medium. Fluorescent labeling with membrane-permeable **SiR-Tet** (magenta) serves as positive control of transfection. Scale bar: 20 µm; Spectral detection: (**SiR-Tet**); λ_exc_: 638 nm/λ_em_: 650–800 nm; dyes **CBRD2-BAT** and **CBRD4-BAT**: λ_exc_: 552 nm/λ_em_: 565–800 nm. Objective: 40×.

**Figure 4:**
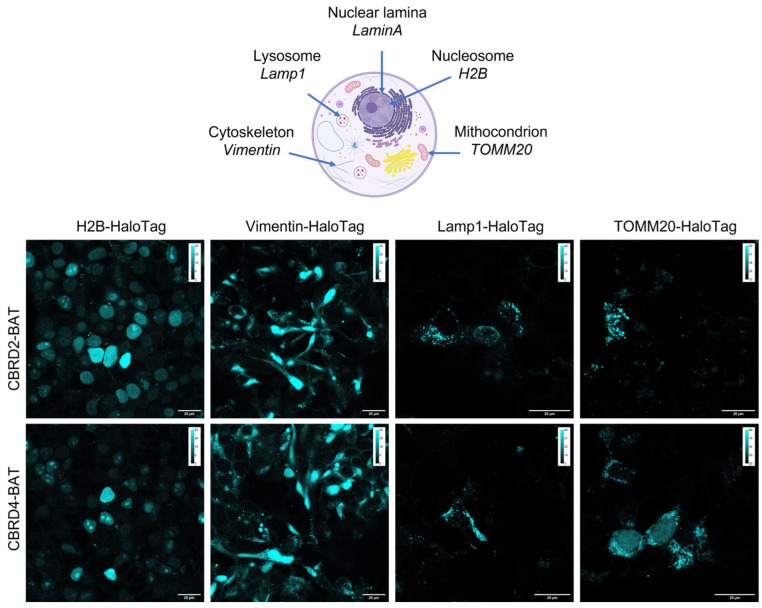
Confocal microscopy images of H2B-, Vimentin-, Lamp1-, TOMM20-HaloTag expressing HEK-293-OATP3A1 cells pretreated with HaloBCN (3 µM, 60 min) and treated with non-permeable fluorescent dyes **CBRD2-BAT** (3 µM, 30 min) and **CBRD4-BAT** (10 µM, 30 min) (cyan) in complete DMEM medium (for images of mock cells cf SI Figures S6–S9). Scale bar: 20 µm; Spectral detection: **CBRD2-BAT** and **CBRD4-BAT**; λ_exc_: 552 nm/λ_em_: 565–800 nm. Objective: 100×.

**Figure 5:**
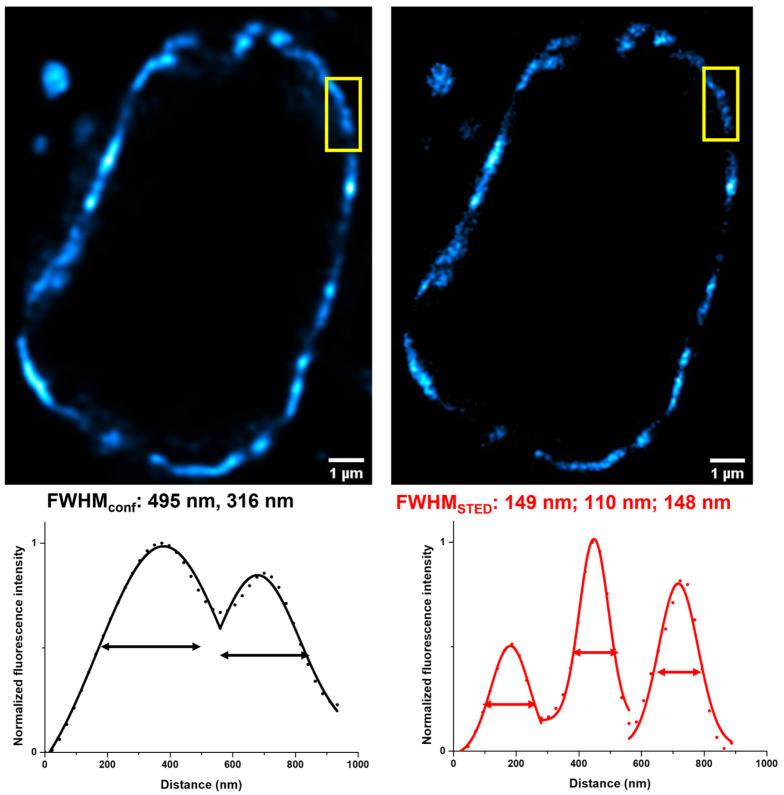
Confocal (left) and super-resolution (STED) (right) microscopy images of Lamin-HaloTag expressing HEK-293-OATP3A1 cells pretreated with HaloBCN (3 µM, 60 min) and treated with non-permeable fluorescent dye **CBRD4-BAT** (10 µM, 30 min) (cyan) in complete DMEM medium. Scale bar: 1 µm; Spectral detection: **CBRD4-BAT**; λ_exc_: 552 nm/λ_em_: 565–800 nm; depletion laser: 660 nm; Objective: 100×. Line diagram of the indicated area is characterized with full width at half maxima values (FWHM).

First, Lamin-HaloTag expressing HEK-293-OATP3A1 and mock cells were treated with HaloBCN to install the complementary bio-orthogonal function (i.e., cyclooctyne, BCN) onto the protein. The as-treated cells were then allowed to react with the **CBRD-BAT** dyes, resulting in intensive specific fluorescence in the nuclear membrane in HEK-293-OATP3A1 cells treated either with **CBRD2-BAT** or **CBRD4-BAT** (Figure 3). To find the optimal conditions for labeling, different concentrations (1, 3, 6 and 10 µM) and incubation times (15, 30 and 60 min) were studied for **CBRD2-BAT** (ESI Figure S4). Evaluation of the data suggested that we use the probe at 3 µM concentration with 30 min incubation time in DMEM. Considering the higher K_M_ value and viability, 10 µM concentration was selected for **CBRD4-BAT**. Satisfyingly, no fluorescence could be seen in case of mock cells. Treatment of both cell-lines (i.e., HEK-293-OATP3A1 or mock) expressing bio-orthogonalized LaminA with a membrane permeable, tetrazine bearing silicon rhodamine (**SIR-Tet**) probe [30], on the other hand, resulted in intensive specific labeling of the nuclear membrane, confirming bio-orthogonal reaction-based staining (Figure 3). The similar labeling efficiency of mock and HEK-293 OATP3A1 cells with SIR-Tet also confirms similar expression of LaminA-HaloTag in the two cell lines. A comparably excellent fluorescent staining of LaminA was achieved with **CBRD4-BAT** (10 µM, 30 min) dye in HEK-293-OATP3A1 cells in DMEM, as well (Figure 3). Satisfyingly, we experienced likewise results in case of further intracellular proteins, i.e., H2B, vimentin, Lamp1 and TOMM20 involved in this study, in complete DMEM medium (Figure 4, and ESI, Figures S5–S9). Formerly, we have proven the suitability of **CBRD4**-core in STED microscopy [28]; thus, we subjected **CBRD4-BAT** tagged proteins to STED microscopy imaging (λ_exc_: 552 nm/λ_em_: 565–800 nm; depletion laser: 660 nm). We have tested live and LaminA-HaloTag expressing HEK-293-OATP3A1 cells in complete DMEM medium and obtained improved spatial resolution of the stained structure using STED microscopy (Figure 5).

## 3. Materials and Methods

### 3.1. General

Synthesis of **CBRD2-BAT** and **CBRD4-BAT** was described earlier [28]. Benzbromarone was purchased from Sigma-Aldrich (Sigma, Merck, Budapest, Hungary).

### 3.2. Cell Cultures

The original HEK-293 cell line was obtained from ATCC. OATP3A1 overexpression in HEK-293 cells was achieved by recombinant lentiviruses. Retroviral transductions of HEK-293 cells were performed as described earlier [16]. In order to generate mock cells, HEK-293 cells were transduced with lentiviruses containing only the pRRL-EF1- ΔCD4 vector [16].

Cells were maintained in Dulbecco’s modified Eagle’s medium (DMEM, Life Technologies 41965-039). The medium was supplemented with 1% penicillin-streptomycin (Gibco 15140-122), 1% Glutamax (Gibco 35050-061), 1% sodium pyruvate (Life Technologies 11360-070), and 10% FBS (Gibco 10500-064). The cells were cultured at 37 °C in a 5% CO_2_ atmosphere and passaged using trypsin (Gibco 25300-054) every 3–4 days up to 20 passages.

### 3.3. Western Blot Analysis

Expression of OATP3A1 was confirmed by Western blot as described earlier [14]. Whole-cell lysates of HEK-293 cells were separated on 7.5% SDS/PAGE gels and transferred onto PVDF membranes. OATP3A1 was detected by using an anti-OATP3A1 (anti-SLCO3A1, SAB 1304633, Sigma Aldrich (Sigma, Merck, Budapest, Hungary)). As a secondary antibody, the peroxidase-conjugated anti-rabbit antibody (Jackson ImmunoResearch (Cambridgeshire, UK)) was used in a dilution of 20,000×. Proteins were visualized using Luminor Enhancer Solution kit by Thermo Fisher Scientific (Waltham, MA, USA).

### 3.4. Transport Activity Measurements by Flow Cytometry

HEK-293 cells overexpressing OATP3A1 and mock cells were trypsinized and collected from a 75cm^2^ culture flask. After washing twice in Uptake buffer (25 mM MES, 125 mM NaCl, 4.8 mM KCl, 1.2 mM CaCl_2_, 1.2 mM KH_2_PO_4_, 12 mM MgSO_4_, and 5.6 mM glucose; pH 5.5 or 7.4) or in complete DMEM medium, 5 × 10^5^ cells/sample (each in triplicate) in 50 µL reaction buffer were pre-incubated for 15 min at 37 °C in the presence or absence of the inhibitor benzbromarone (final concentration 20–80 µM; the exact concentration indicated at Figure legends). Transport reaction was initiated with the addition of two-times concentrated fluorescent CBRD-BAT compounds (final concentration of 0.1–30 µM; the exact concentrations are indicated in the Figure legends). Cells were incubated with the compounds for 15 min at 37 °C. Uptake was stopped by adding 400 µL ice-cold PBS.

The fluorescence (mean RFU) of at least 10,000 cells was determined within an hour using an Attune NxT Flow Cytometer (Life Technologies, Carlsbad, CA, USA). Data were handled by using the Attune NxT Software v3.1.2. Cells were gated to single cells after excluding debris and aggregates. Fluorescence was acquired with a blue laser excitation (488 nm) in the BL3 detection channel (695/40).

Transport activity was normalized to the background levels, i.e., to fluorescence measured in mock cells (transduced with empty lentivirus vector).

For data analysis, GraphPad Prism 8 software was used to determine non-linear curve fitting (Michaelis–Menten equation).

### 3.5. Effect of Dyes on Cell Viability

A viability test was carried out to assess the toxicity of CBRD-BAT probes on HEK-293-OATP3A1 and mock cells as well. Cells (30,000 cells/well) were transferred into a 48-well plate (Thermo Fisher Scientific, 130187)—coated for 4h with 0.01 mg/mL Poly-L-lysine (Sigma P5899)—and were incubated for 48 h at 37 °C in a 5% CO_2_ atmosphere. After that period, DMEM culture medium was exchanged either to fresh DMEM medium or to Uptake buffer pH 5.5. Cells were treated with CBRD-BAT dyes in the respective media (Uptake or DMEM) as follows: 1 µM for 15 min for both dyes in Uptake, or 3 µM and 10 µM for CBRD2-BAT and CBRD4-BAT, respectively, for 30 min in DMEM at 37 °C in the dark, according to the applied incubation concentrations and periods for sample treatment for microscopy analyses. This step was followed by slight washing with complete DMEM and a 2 h incubation period (conciliated with the duration of live cell labeling) at 37 °C in 5% CO_2_ atmosphere. After the incubation period, supernatants were replaced with 0.5 mg/mL MTT (3-(4,5-dimethylthiazol-2-yl)-2,5-diphenyltetrazolium bromide) solution (in complete DMEM) and incubated for 120 min at 37 °C in the dark. The insoluble formazan crystals were dissolved in 250 µL DMSO. Absorbance was detected at 540 nm using a Biotek Synergy 2 Cytation 3 imaging plate reader with Gen5 software version 3.08 (Biotek, Winooski, VT, USA). Viability was expressed as percentage (n = 3) of the readings of untreated control cells.

### 3.6. Live-Cell Labeling

HEK-293-OATP3A1 and mock (40,000 cell/well) cells were transferred into µ-Slide 8 well plates (Ibidi 80827) and were incubated for 40 h at 37 °C in a 5% CO_2_ atmosphere. Ibidi plates were pretreated with 0.01 mg/mL Poly-L-lysine (Sigma P5899) for 4 h at room temperature and washed afterwards. Cells were transfected with 0.25 μg Lamin-HaloTag, H2B-HaloTag, Vimentin-HaloTag, Lamp1-HaloTag or TOMM20-HaloTag [31] plasmid using JetPrime (Polyplus 114-07) transfection agent for four hours according to the manufacturer’s protocol. Subsequently, the supernatant was replaced with a fresh medium for overnight. One day after transfection, the bio-orthogonally reactive chemical reporter BCN was administered in HaloTag substrate (HaloBCN) [31] (cf. Sceme S1) in concentration of 3 µM for 1h. After replacing the medium, cells were labeled with the fluorescent dyes CBRD2-BAT and CBRD4-BAT at a concentration of 3 µM and 10 µM in complete DMEM medium, respectively, for 30 min at 37 °C in the dark. Afterwards, a two-hour washing step with indicator-free complete DMEM was interpolated followed by fixation (4% formaldehyde for 10 min at 25 °C) and quick washing—twice—with PBS prior to imaging.

### 3.7. Confocal and STED Imaging and Analysis

Confocal and STED images were acquired on a Leica TCS SP8 STED 3X microscope using the 552 nm laser for excitation and 660 nm STED (1.5 W, continuous wave) laser for depletion. The images were taken using a Leica HC PL APO 100×/1.40 or 40×/1.3 oil immersion objective using the Leica HyD detector.

Applying the Huygens Professional software (SVI), we performed deconvolution for image restoration on the recorded STED images. The deconvolution was based on theoretical point spread function (PSF). Images were analyzed using Leica Application Suite X and ImageJ software (NIH). We selected the results of some representative line analysis for demonstration. Non-linear Gaussian curve was fitted to the normalized fluorescence intensity values by using Origin Pro 9 software. For characterizing the resolution efficiency, the full width at half maximum values (FWHM) were given. For calculation of fluorescence signal ratio of fluorescently labeled HEK-293-OATP3A1 and mock cells was calculated with Fiji for Image J software (NIH). Integrated signal of the image area was determined.

## 4. Conclusions

Our recent findings on the specific transport of non-permeable zwitterionic dyes by organic anion transporting polypeptide 3A1 provoked the delineation of an OATP3A1 gated highly specific bio-orthogonal labeling scheme. To this end, we first confirmed the transport of two membrane impermeable, bio-orthogonally applicable, tetrazine bearing, large Stokes shift dyes (CBRD2-BAT and CBRD4-BAT) by OATP3A1 expressing cells at two different transport conditions. We concluded that transport of the dyes is highly efficient in Uptake buffer pH 5.5 but also satisfying in complete DMEM medium, which is more suitable for live-cell studies. We also tested the performance of the envisioned OATP3A1 assisted bio-orthogonal labeling schemes in the specific tagging of various intracellular proteins modified genetically with HaloTag. Installation of the complementary bio-orthogonal function onto the protein of interest was effected by treatment of the HaloTagged proteins with a cyclooctynylated HaloTag substrate, HaloBCN. Treatment of the as-prepared live cells with CBRD-BAT probes resulted in highly specific fluorescent tagging of the proteins in each case when OATP3A1 was present, while no labeling could be seen in the absence of the transport protein. We have also performed STED imaging of CBRD4-BAT stained protein structures.

We believe that the presented OATP3A1 conditioned bio-orthogonal labeling scheme using non-permeable fluorescent dyes paves the way to systematically screen for proteins whose expression is linked to OATP3A1 activity. Such studies will certainly facilitate the better understanding of this less studied transport protein.

## Data Availability

The authors confirm that the generated data are available in the manuscript.

## Supplementary Materials

The following supporting information can be downloaded at: https://www.mdpi.com/article/10.3390/molecules28062521/s1, Figure S1. Western blot detection of OATP3A1 expressed in HEK-293-OATP3A1 and mock cells; Figure S2. Concentration dependence of influx (mean RFU) of dyes CBRD2-BAT and CBRD4-BAT; Figure S3. The effect of the Uptake buffer pH 5.5 and complete DMEM media and the bio-orthogonal dyes on the viability of the HEK-293-OATP3A1 cells compared to the mock ones; Scheme S1. Scheme of sequential bio-orthogonal fluorescent labeling through self-labeling enzyme Tag (HaloTag) fused to POI (protein of interest) with HaloBCN and tetrazine modified fluorescent dye; Figure S4. Confocal microscopy images of Lamin-HaloTag expressing HEK-293-OATP3A1 cells pretreated with HaloBCN and treated with non-permeable fluorescent dye CBRD2-BAT; Figures S5–S9. Confocal microscopy images of POI-HaloTag expressing HEK-293-OATP3A1 (OATP3A1) and mock cells pretreated with HaloBCN and treated with non-permeable fluorescent dyes CBRD2-BAT and CBRD4-BAT.

## Sample Availability

Samples of the compounds are available from the authors.
