# Supplementary material for: Organic Anion Transporting Polypeptide 3A1 (OATP3A1)-Gated Bio-Orthogonal Labeling of Intracellular Proteins
Source: Molecules. 2023 Mar 9;28(6):2521. doi: 10.3390/molecules28062521 (PMC10055104; doi:10.3390/molecules28062521)
Supplement: Supplementary file 1 [file molecules-28-02521-s001.zip › molecules-2258672-supplementary.pdf]

Supporting Information for
Organic anion transporting polypeptide 3A1 (OATP3A1) gated bioorthogonal labeling of intracellular proteins

Krisztina Németh [1],*, Zsófia László [1], Adrienn Biró [1], Ágnes Szatmári [1], Gergely B. Cserép [1], György Várady [2], Éva Bakos [3], Csilla Özvegy-Laczka [3], Péter Kele [1],*

[1] Chemical Biology Research Group, Institute of Organic Chemistry, RCNS, Magyar tudósok krt. 2., H-1117 Budapest, Hungary;

[2] Molecular Cell Biology Research Group, Institute of Enzymology, RCNS, Magyar tudósok krt. 2., H-1117 Budapest, Hungary;

[3] Membrane Protein Research Group, Institute of Enzymology, RCNS, Magyar tudósok krt. 2., H-1117 Budapest, Hungary;

* Correspondence: nemeth.krisztina@ttk.hu and kele.peter@ttk.hu

Contents

1. OATP3A1 protein expression in HEK-293-OATP3A1 and mock cells 2

2. Influx kinetics of the dyes **CBRD2-BAT** and **CBRD4-BAT** 2

3. Effect of dyes on cell viability 3

4. Live-cell labeling 4

*4.1. Optimization of labeling concentration and time with dyes **CBRD2-BAT** and **CBRD4**-BAT* .5

4.2. Confocal microscopy imaging of bioorthogonal labeled intracellular POIs with dyes CBRD2-BAT and CBRD4-BAT in complete DMEM 6

1. OATP3A1 protein expression in HEK-293-OATP3A1 and mock cells

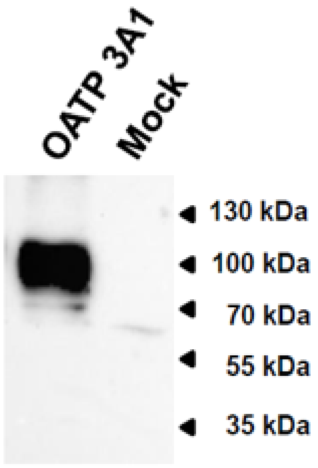

Figure S1. Western blot detection of OATP3A1 expressed in HEK-293-OATP3A1 and mock cells. 5 µg of total cell lysates were analyzed. OATP3A1 was detected by an anti-OATP3A1 antibody. Experiments were repeated three times and one representative Western blot is shown.

2. Influx kinetics of the dyes **CBRD2-BAT** and **CBRD4-BAT**

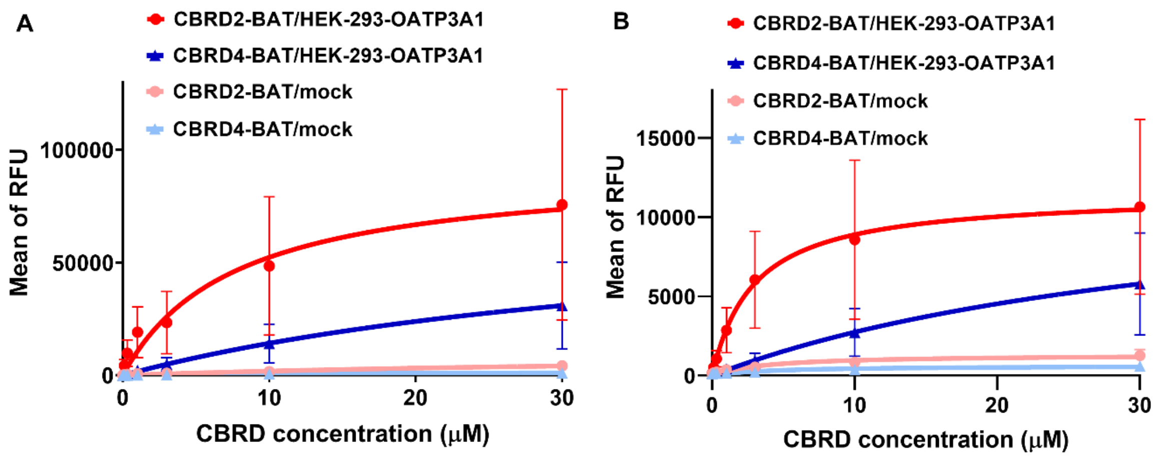

Figure S2. Concentration dependence of influx (mean RFU) of dyes **CBRD2-BAT** and **CBRD4-BAT** into OATP3A1 expressing and mock cells in Uptake buffer pH 5.5 (A) and in complete DMEM medium (B) determined with flow cytometry. K_m values are 7.2 µM and 45 µM for **CBRD2-BAT** and **CBRD4-BAT**, respectively in Uptake buffer; 2.9 µM and 50.2 µM for **CBRD2-BAT** and **CBRD4-BAT**, respectively in complete DMEM. Transport data were obtained by subtracting the fluorescence (RFU) in mock cells

3. Effect of dyes on cell viability

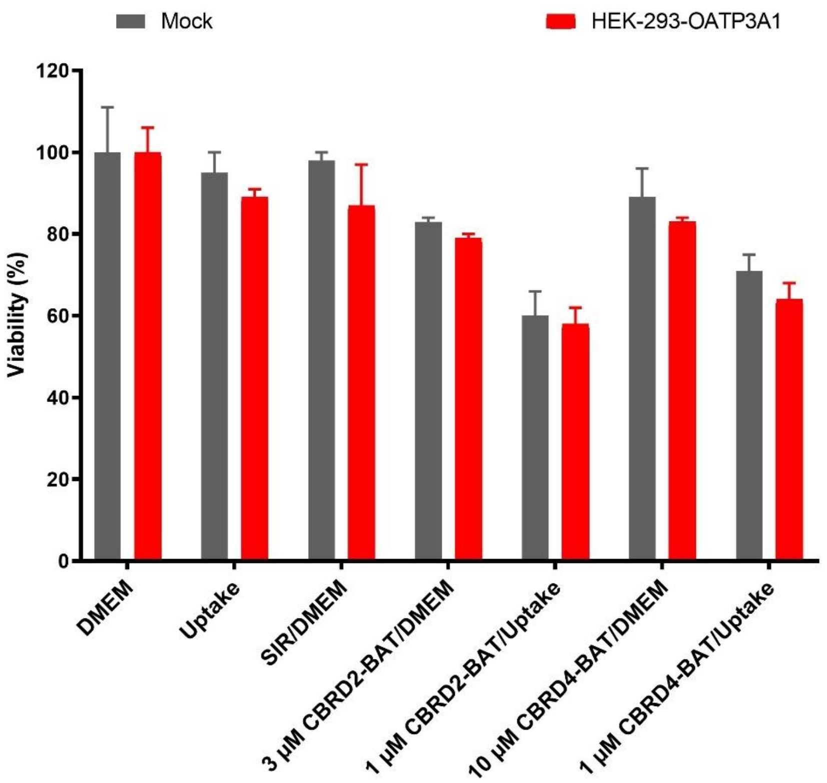

Figure S3. The effect of the Uptake buffer pH 5.5 and complete DMEM media and the bioorthogonal dyes on the viability of the HEK-293-OATP3A1 cells compared to the mock ones

4. Live-cell labeling

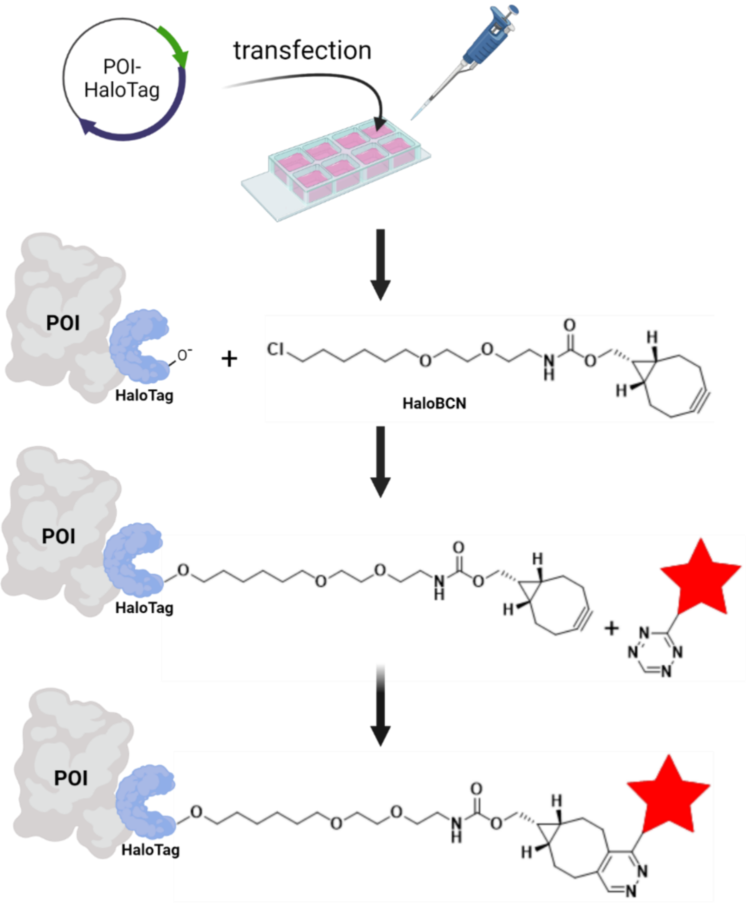

Scheme S1. Scheme of sequential bioorthogonal fluorescent labeling through self-labeling enzyme Tag (HaloTag) fused to POI (protein of interest) with HaloBCN and tetrazine modified fluorescent dye. Created with BioRender.com

4.1. *Optimization of labeling concentration and time with dyes* ***CBRD2-BAT*** *and* ***CBRD4-BAT***

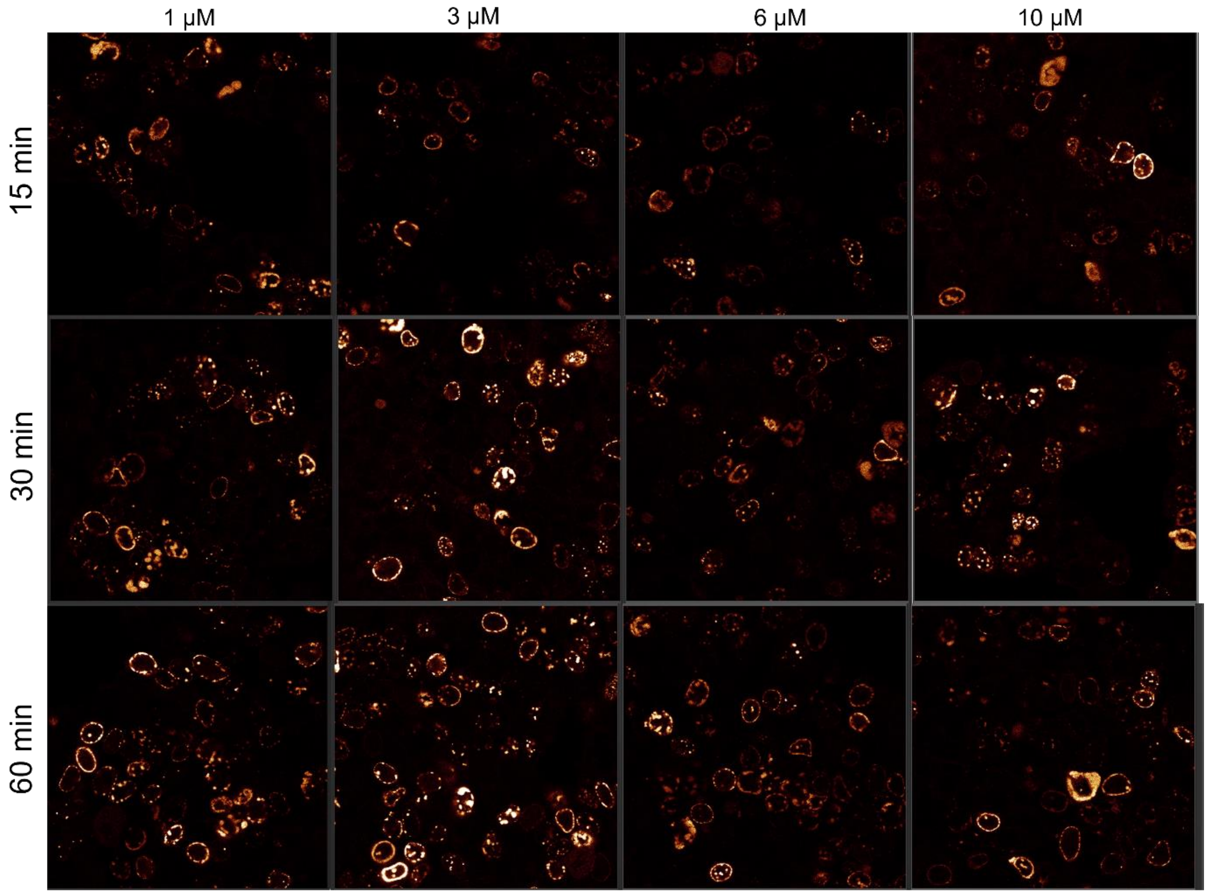

Figure S4. Confocal microscopy images of Lamin-HaloTag expressing HEK-293-OATP3A1 cells pretreated with HaloBCN (3 µM, 60 min) and treated with non-permeable fluorescent dye **CBRD2-BAT** (1-3-6-10 µM, 15-30-60 minutes) in complete DMEM medium. Spectral detection: λ_{exc}: 552 nm / λ_{em}: 565-800 nm. Objective: 40x.

4.2. *Confocal microscopy imaging of bioorthogonal labeled intracellular POIs with dyes **CBRD2-BAT** and **CBRD4-BAT** in complete DMEM*

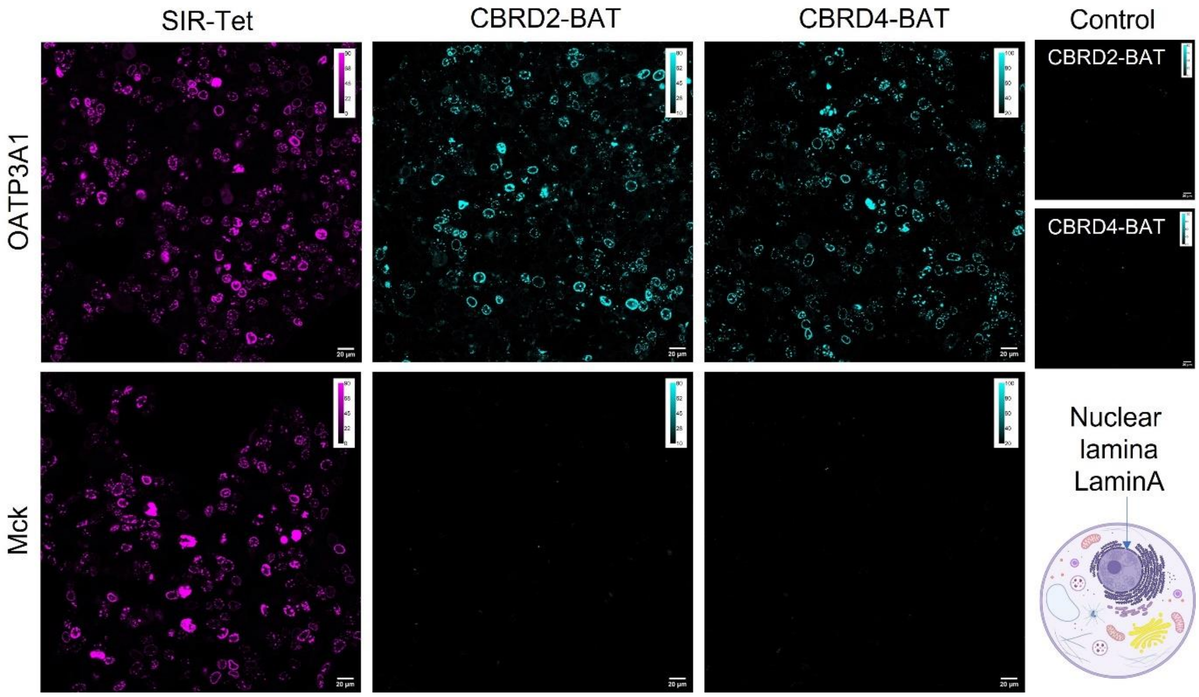

Figure S5. Confocal microscopy images of LaminA-HaloTag expressing HEK-293-OATP3A1 (OATP3A1) and mock cells pretreated with HaloBCN (3 µM, 60 min) and treated with non-permeable fluorescent dyes **CBRD2-BAT** (3 µM, 30 min) and **CBRD4-BAT** (10 µM, 30 min) (cyan) in complete DMEM medium. Fluorescent labeling with membrane-permeable **SiR-Tet** (3 µM, 60 min) (magenta) serves as positive control of transfection. Cells without transfection (Contol) shows the background fluorescent signals of **CBRD2-BAT** (3 µM) and **CBRD4-BAT** (10 µM) in OATP3A1 cells. Scale bar: 20 µm. Spectral detection: (SiR-Tet): λ_{exc}: 638 nm / λ_{em}: 650-800 nm; dyes **CBRD2-BAT** and **CBRD4-BAT**: λ_{exc}: 552 nm / λ_{em}: 565-800 nm. Objective: 40x.

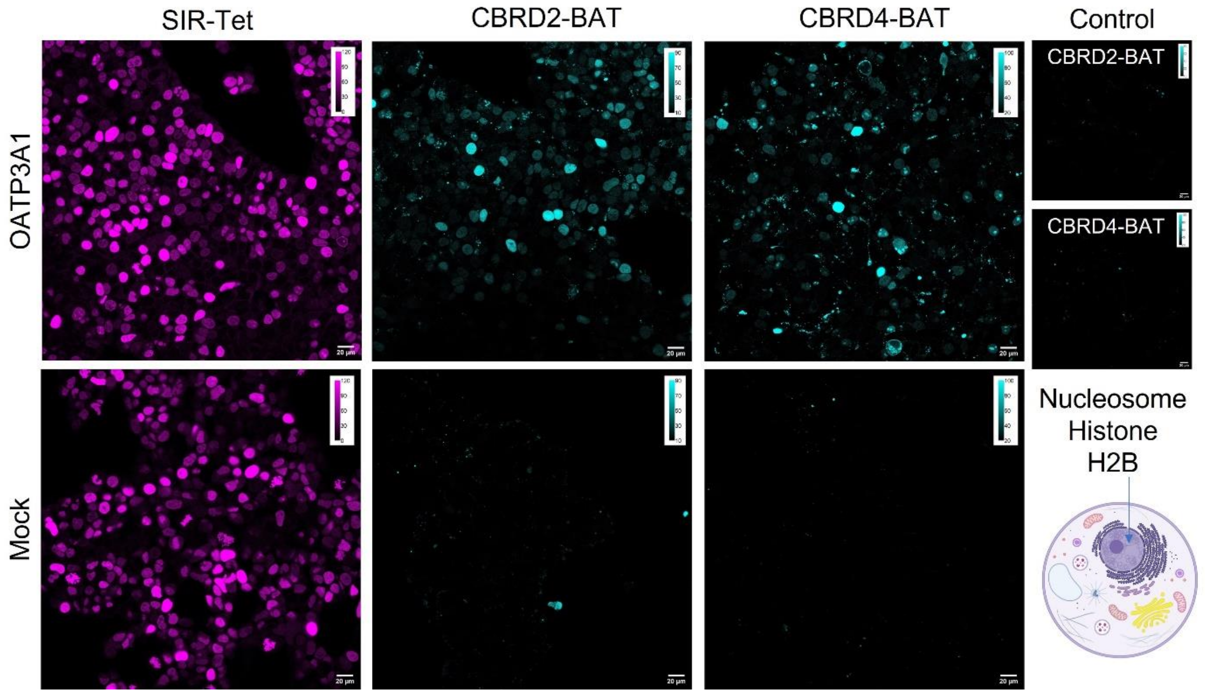

Figure S6. Confocal microscopy images of H2B-HaloTag expressing HEK-293-OATP3A1 (OATP3A1) and mock cells pretreated with HaloBCN (3 µM, 60 min) and treated with non-permeable fluorescent dyes **CBRD2-BAT** (3 µM, 30 min) and **CBRD4-BAT** (10 µM, 30 min) (cyan) in complete DMEM medium. Fluorescent labeling with membrane-permeable **SiR-Tet** (3 µM, 60 min) (magenta) serves as positive control of transfection. Cells without transfection (Contol) shows the background fluorescent signals of **CBRD2-BAT** (3 µM) and **CBRD4-BAT** (10 µM) in OATP3A1 cells. Scale bar: 20 µm. Spectral detection: (SiR-Tet): λ_{exc}: 638 nm / λ_{em}: 650-800 nm; dyes **CBRD2-BAT** and **CBRD4-BAT**: λ_{exc}: 552 nm / λ_{em}: 565-800 nm. Objective: 40x.

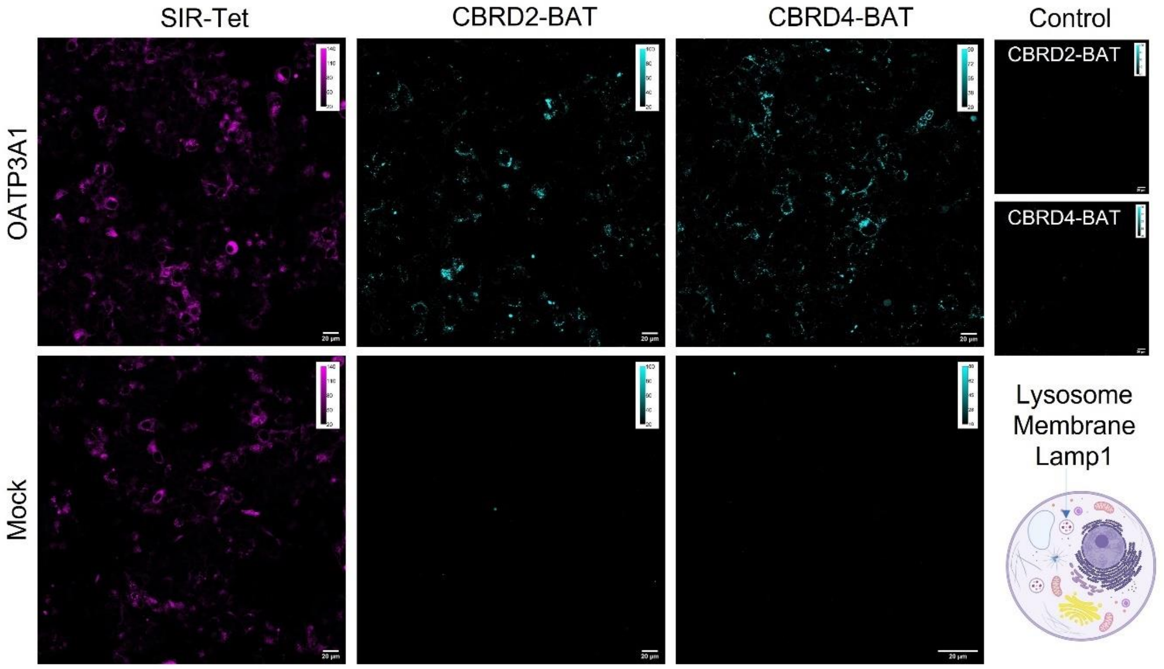

Figure S7. Confocal microscopy images of Lamp1-HaloTag expressing HEK-293-OATP3A1 (OATP3A1) and mock cells pretreated with HaloBCN (3 µM, 60 min) and treated with non-permeable fluorescent dyes **CBRD2-BAT** (3 µM, 30 min) and **CBRD4-BAT** (10 µM, 30 min) (cyan) in complete DMEM medium. Fluorescent labeling with membrane-permeable **SiR-Tet** (3 µM, 60 min) (magenta) serves as positive control of transfection. Cells without transfection (Contol) shows the background fluorescent signals of **CBRD2-BAT** (3 µM) and **CBRD4-BAT** (10 µM) in OATP3A1 cells. Scale bar: 20 µm. Spectral detection: (SiR-Tet): λ_{exc}: 638 nm / λ_{em}: 650-800 nm; dyes **CBRD2-BAT** and **CBRD4-BAT**: λ_{exc}: 552 nm / λ_{em}: 565-800 nm. Objective: 40x.

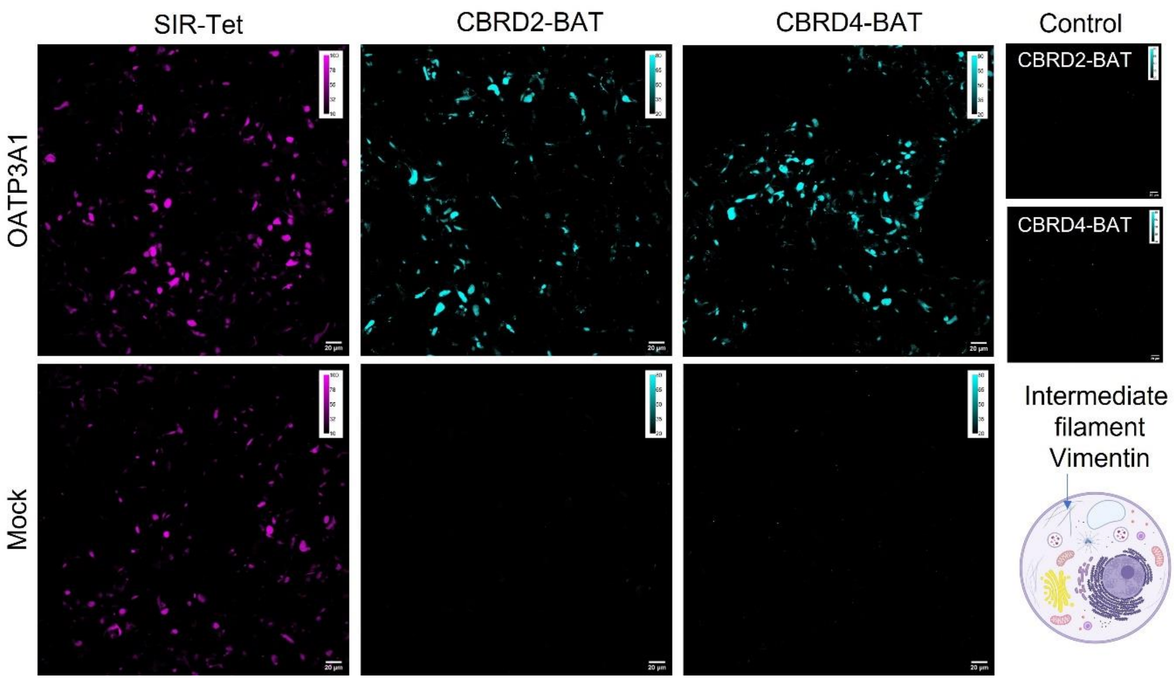

Figure S8. Confocal microscopy images of Vimentin-HaloTag expressing HEK-293-OATP3A1 (OATP3A1) and mock cells pretreated with HaloBCN (3 µM, 60 min) and treated with non-permeable fluorescent dyes **CBRD2-BAT** (3 µM, 30 min) and **CBRD4-BAT** (10 µM, 30 min) (cyan) in complete DMEM medium. Fluorescent labeling with membrane-permeable **SiR-Tet** (3 µM, 60 min) (magenta) serves as positive control of transfection. Cells without transfection (Contol) shows the background fluorescent signals of **CBRD2-BAT** (3 µM) and **CBRD4-BAT** (10 µM) in OATP3A1 cells. Scale bar: 20 µm. Spectral detection: (SiR-Tet): λ_{exc}: 638 nm / λ_{em}: 650-800 nm; dyes **CBRD2-BAT** and **CBRD4-BAT**: λ_{exc}: 552 nm / λ_{em}: 565-800 nm. Objective: 40x.

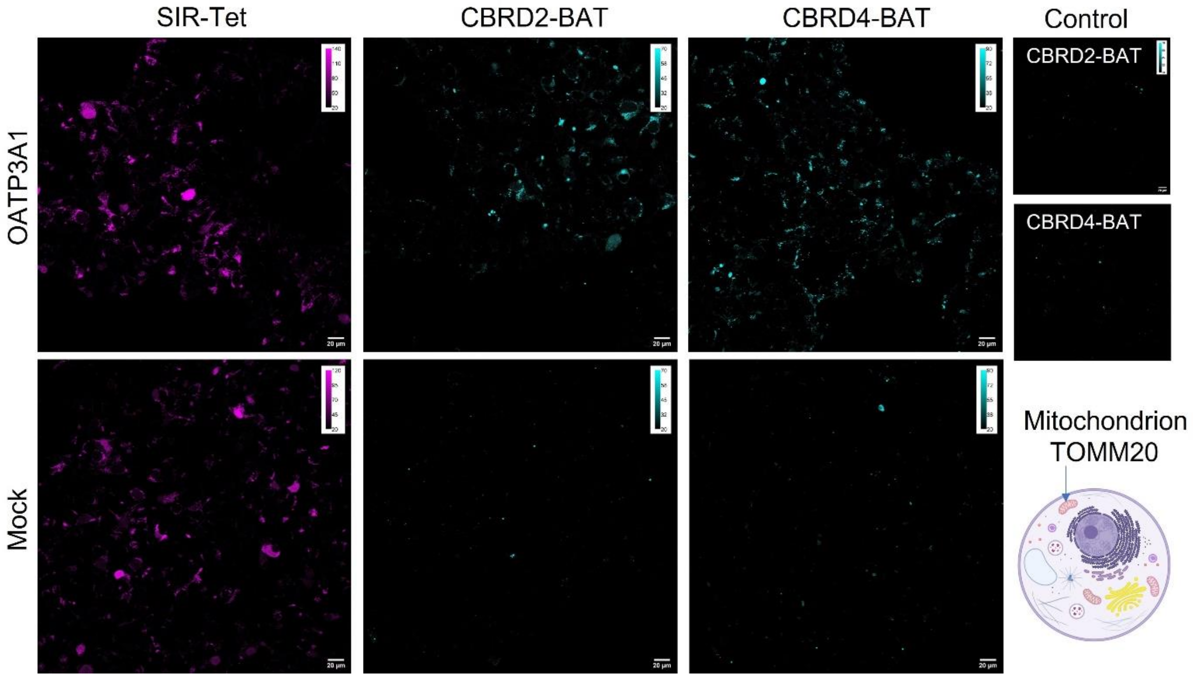

Figure S9. Confocal microscopy images of TOMM20-HaloTag expressing HEK-293-OATP3A1 (OATP3A1) and mock cells pretreated with HaloBCN (3 µM, 60 min) and treated with non-permeable fluorescent dyes **CBRD2-BAT** (3 µM, 30 min) and **CBRD4-BAT** (10 µM, 30 min) (cyan) in complete DMEM medium. Fluorescent labeling with membrane-permeable SiR-Tet (3 µM, 60 min) (magenta) serves as positive control of transfection. Cells without transfection (Contol) shows the background fluorescent signals of **CBRD2-BAT** (3 µM) and **CBRD4-BAT** (10 µM) in OATP3A1 cells. Scale bar: 20 µm. Spectral detection: (SiR-Tet): λ_{exc}: 638 nm / λ_{em}: 650-800 nm; dyes **CBRD2-BAT** and **CBRD4-BAT**: λ_{exc}: 552 nm / λ_{em}: 565-800 nm. Objective: 40x.
